# Exosomes from Human Adipose Tissue-Derived Mesenchymal Stem Cells Promote Epidermal Barrier Repair by Inducing de Novo Synthesis of Ceramides in Atopic Dermatitis

**DOI:** 10.3390/cells9030680

**Published:** 2020-03-10

**Authors:** Kyong-Oh Shin, Dae Hyun Ha, Jin Ock Kim, Debra A. Crumrine, Jason M. Meyer, Joan S. Wakefield, Yerin Lee, Bogyeong Kim, Sungeun Kim, Hyun-keun Kim, Joon Lee, Hyuck Hoon Kwon, Gyeong-Hun Park, Jun Ho Lee, Jihye Lim, Sejeong Park, Peter M. Elias, Kyungho Park, Yong Weon Yi, Byong Seung Cho

**Affiliations:** 1 Department of Food Science and Nutrition, Convergence Program of Material Science for Medicine and Pharaceutics, Hallym University, Chuncheon 24252, Korea; 0194768809@hanmail.net (K.-O.S.); dldpfls1352@naver.com (Y.L.); bokyung0725@naver.com (B.K.); sungeuna27@naver.com (S.K.); 2 ExoCoBio Exosome Institute (EEI), ExoCoBio Inc., Seoul 08594, Korea; dh.ha@exocobio.com (D.H.H.); jinock@gmail.com (J.O.K.); hyunkeun.kim@exocobio.com (H.-k.K.); junho.lee@exocobio.com (J.H.L.); Jihye.lim@exocobio.com (J.L.); sejeong.park@exocobio.com (S.P.); 3 Department of Dermatology, University of California, NCIRE, and Veterans Affairs Medical Center, San Francisco, CA 94121, USA; debcrumrine@yahoo.com (D.A.C.); jmmeye3@gmail.com (J.M.M.); Joan.Wakefield@ucsf.edu (J.S.W.); peter.elias@ucsf.edu (P.M.E.); 4 School of Chemical and Biological Engineering, Seoul National University, Seoul 151-744, Korea; purequill@naver.com; 5 Oaro Dermatology Clinic, Seoul 13620, Korea; banbury@hanmail.net; 6 Department of Dermatology, Dongtan Sacred Heart Hospital, Hallym University College of Medicine, Hwaseong-si, Gyeonggi-do 431-060, Korea; jin66666@hanmail.net

**Keywords:** ASC-exosomes, anti-inflammation, atopic dermatitis, restoration, skin barrier, lamella body, ceramides

## Abstract

Atopic dermatitis (AD) is a multifactorial, heterogeneous disease associated with epidermal barrier disruption and intense systemic inflammation. Previously, we showed that exosomes derived from human adipose tissue-derived mesenchymal stem cells (ASC-exosomes) attenuate AD-like symptoms by reducing multiple inflammatory cytokine levels. Here, we investigated ASC-exosomes’ effects on skin barrier restoration by analyzing protein and lipid contents. We found that subcutaneous injection of ASC-exosomes in an oxazolone-induced dermatitis model remarkably reduced trans-epidermal water loss, while enhancing stratum corneum (SC) hydration and markedly decreasing the levels of inflammatory cytokines such as IL-4, IL-5, IL-13, TNF-α, IFN-γ, IL-17, and TSLP, all in a dose-dependent manner. Interestingly, ASC-exosomes induced the production of ceramides and dihydroceramides. Electron microscopic analysis revealed enhanced epidermal lamellar bodies and formation of lamellar layer at the interface of the SC and stratum granulosum with ASC-exosomes treatment. Deep RNA sequencing analysis of skin lesions demonstrated that ASC-exosomes restores the expression of genes involved in skin barrier, lipid metabolism, cell cycle, and inflammatory response in the diseased area. Collectively, our results suggest that ASC-exosomes effectively restore epidermal barrier functions in AD by facilitating the de novo synthesis of ceramides, resulting in a promising cell-free therapeutic option for treating AD.

## 1. Introduction

Atopic dermatitis (AD) is a chronic, relapsing, and highly pruritic inflammatory skin disease that significantly reduces the quality of life [1,2] for patients. For many years, the pathogenesis and development of AD have been mostly attributed to the T helper (Th) type 1/Th type 2 immune dysregulation (inside–outside hypothesis). Accordingly, most therapies have been focused on reducing Th2-mediated immune responses [3]. However, increasing evidence has also pointed to a critical role of structural abnormalities in the stratum corneum (SC), related to the outside–inside hypothesis, in AD pathophysiology [4,5]. Although the relationship between the epidermal barrier defect and immunological abnormalities in the pathogenesis of AD still needs to be elucidated, recent studies have shown the strong synergism between dysregulation of immune response such as elevated Th2 cytokine levels and abnormal expression of genes responsible for epidermal barrier functions to increase susceptibility to AD [6]. The data indicate that both defective skin barrier and abnormal immune responses are crucial factors in the development of AD and therefore, the multi-pronged approach, which involves repair of leaky skin barriers as well as suppression of skin inflammation, is essential for the effective treatment of AD.

Mesenchymal stem cells (MSCs) are multipotent stem cells that reside in adult tissues. Because MSCs can differentiate into many cell types including adipocytes, osteoblasts, and chondrocytes [7], their multi-potentiality and unique ability to self-renew have made them a promising cell-based strategy to treat several diseases. Among them, adipose tissue-derived MSCs (ASCs) have several advantages compared to other types of adult stem cells since it is relatively easy to obtain them, they are available in large amounts with minimal morbidity through liposuction, and the isolation procedure is simple [8]. Moreover, ASCs have a unique immune modulatory function [9,10], which makes them suitable for cellular therapy, such as repairing tissue damaged by chronic inflammation or autoimmune diseases. For example, it has been reported that the transplantation of ASCs exerts beneficial effects in murine models of experimental autoimmune encephalomyelitis (EAE) [11], systemic lupus erythematous (SLE) [12], and autoimmune diabetes [13]. However, there have also been several drawbacks limiting their therapeutic benefits which include poor engraftment efficiency, non-specific differentiation, potential risk of tumor formation, short half-life, and difficulty of quality controls [14].

Extracellular vesicles (EVs) are defined as lipid bilayered vesicles shed by all most all cells toward extracellular space and include exosomes, (30–200 nm in diameter), microvesicles (200–1000 nm in diameter), and apoptotic bodies (500–2000 nm in diameter) [15]. Initially, exosomes were thought to be metabolic byproducts of cells to dispose of cellular waste [16], but now it is widely established that exosomes serve as essential mediators of intracellular communication by transferring proteins and genetic materials such as mRNA, microRNA (miRNA), and DNA [17]. Recently, evidence has indicated that MSC-derived exosomes (MSC-exosomes) play a critical role in mediating the beneficial effects of MSCs in tissue regeneration [18]. For example, administration of MSC-derived exosomes showed therapeutic potential in cardiovascular diseases including myocardial infarction [19], reperfusion injury [20], and pulmonary hypertension [21]. It was also shown that MSC-exosomes suppress graft-vs.-host-disease (GvHD) symptoms by mimicking the therapeutic effects of the MSCs treatment [22]. These data suggest that exosomes could be a compelling alternative to MSCs in regenerative medicine because they would help avoid most of the problems involved with using live MSC-based therapy [23].

Previously, we showed that ASC-exosomes ameliorate AD symptoms in vivo by reducing systemic inflammation [24]. However, the therapeutic relevance of ASC-exosomes in restoring normal epidermal barrier function after injury in AD has not yet been tested. Here, we demonstrate that ASC-exosomes are capable of improving epidermal permeability barrier functions, which coincides with a significant increase in ceramides and a reduction in immune responses during the progression of AD. Our findings suggest that ASC-exosomes could be a promising cell-free alternative to current, limited treatment options for AD that have potentially harmful side effects.

## 2. Materials and Methods

### 2.1. Generation of Adipose Tissue-Derived MSCs (ASCs) Conditioned Media

Human adipose tissue from a healthy donor was collected and assessed according to the the Korean Ministry of Food and Drug Safety (MFDS) guidelines. After isolating ASCs from the adipose tissue of a healthy donor, ASCs were sub-cultured at a density of 3000 cells/cm^2^ with Dulbecco’s Modified Eagle’s Medium (DMEM) containing 10% Fetal Bovine Serum (FBS) (Thermo Fisher Scientific, Carlsbad, CA, USA) in a humidified atmosphere of 5% CO_2_ in air at 37 °C. Cells were harvested with Trypsin-EDTA (Thermo Fisher Scientific), to then be washed with DPBS (Thermo Fisher Scientific). Cell stocks of passage 4 were stored in liquid nitrogen (1,100,000 cells/mL/1 vial). The quality of ASCs was assessed by tests for sterility, mycoplasma, cell viability, endotoxin, and viruses. The surface markers for ASCs were determined by flow cytometry against CD29, CD73, CD105, and CD146. Trilineage differentiation (adipogenic, chondrogenic, and osteogenic differentiation) potencies of ASCs were also determined.

To generate the ASC conditioned media (CM), a cell stock was thawed and sub-cultured until passage 7. ASCs at passage 7 were plated at a density of 6000 cells/cm^2^ and cultured with DMEM containing 10% FBS in a humidified atmosphere of 5% CO_2_ in air at 37 °C for 3 days up to 90% confluency. Cells were washed three times with PBS and supplemented with phenol red-free DMEM containing 1% L-Glutamine (200 mM) and 1% Sodium Pyruvate (100 mM) (Thermo Fisher Scientific). The cells were further cultured for 24 h before collecting the CM.

### 2.2. Isolation of ASC-Exosomes

ASC-exosomes (ASCE™, ASCE is the proprietary trademark of ExoCoBio) were isolated from the ASCs CM by the tangential flow filtration (TFF)-based ExoSCRT™ technology as previously described [24]. Briefly, the CM was filtrated through a 0.22-μm polyethersulfone membrane filter (Merck Millipore, Billerica, MA, USA) to remove non-exosomal particles such as cells, cell debris, microvesicles, and apoptotic bodies. The CM was then concentrated by tangential-flow filtration with a 500 kDa molecular weight cut-off filter membrane cartridge (GE Healthcare, Chicago, IL, USA), and buffer exchange was performed by diafiltration with PBS. The amount of proteins in isolated ASC-exosomes was approximately 0.5% of the amount of proteins in the CM. Isolated ASC-exosomes were aliquoted into polypropylene disposable tubes and stored at −80 °C until use. Before using, frozen ASC-Exo were left at 4 °C until completely thawed and were not frozen again. The characterization and profile analysis of ASC-exosomes were conducted following the Minimal Information for Studies of Extracellular Vesicles 2018 (MISEV2018) recommended by the International Society of Extracellular Vesicles (ISEV) [25].

### 2.3. Nanoparticle Tracking Analysis (NTA)

To determine the size distribution and particle concentration, ASC-exosomes diluted with PBS were analyzed by nanoparticle tracking analysis (NTA) using a NanoSight NS300 (Malvern Panalytical, Amesbury, UK) equipped with a 642-nm laser. ASC-exosomes, diluted with PBS to between 20 and 80 particles per frame, were scattered and illuminated by the laser beam and their movements under Brownian motion were captured for 20 s each at a camera level of 16. Videos were then analyzed by the NTA 3.2 software using constant settings. To provide a representative result, at least 5 videos were captured and >2000 validated tracks were analyzed for each individual sample. The NTA instrument was regularly checked with 100 nm-sized standard beads (Thermo Fisher Scientific). To provide representative size distribution of exosomes, size distribution profiles from each video replicates were averaged.

### 2.4. Cryo-Transmission Electron Microscopy (Cryo-TEM)

Cryo-TEM images were obtained using a BIO-TEM installed at the Korea Basic Science Institute (Osong, Korea). The isolated ASC-exosomes were applied to Quantifoil grids (Electron Microscopy Sciences, Hatfield, PA, USA) and subsequently blotted using Vitrobot Mark IV (FEI, Hillsboro, OR, USA) with the following settings: 100% humidity, 4 °C, blot time of 5 s, blot force of 5, blot total of 1, wait time of 5 s, and drain time of 0 s. For maintenance of vitrified sample grids at a temperature of around −178 °C within the TEM, a side entry Gatan 626 cryo-holder (Gatan, Pleasanton, CA, USA) was used. The grids were then examined with a Tecnai G2 Spirit Twin TEM equipped with an anti-contaminator (FEI Company, Hillsboro, OR, USA). A 4K × 4K, Ultrascan 4000 CCD camera (Gatan) was used for image recording. A low-dose method (exposures at 1000 electrons per nm^2^/s) was used for cryo-TEM.

### 2.5. Western Blot Analysis

After lysing ASCs or ASC-exosomes with RIPA buffer (Cell signaling Technology, Danvers, MA, USA), their protein concentrations were determined by the bicinchoninic acid (BCA) protein assay (Thermo Fisher Scientific). Equal amounts of protein samples of ASCs and ASC-exosomes were separated on 10 or 15% SDS-PAGEs and transferred onto polyvinylidene fluoride (PVDF) membranes (EMD Millipore, Billerica, MA, USA). The membranes were blocked with 5% skim milk (BD Biosciences, Franklin, NJ, USA) in TBS-T (0.1% Tween 20 in Tris-buffered saline) for 1 h at room temperature and then incubated with the appropriate primary antibodies against Alix, TSG101, CD9, CD81 (Abcam, Cambridge, MA, USA), GM130 (Cusabio Biotech, Wuhan, China), and Calnexin (Cell Signaling Technology) at 4 °C overnight. After washing, the membranes were incubated with the appropriate horseradish peroxidase (HRP)-conjugated secondary antibodies (Jackson ImmunoResearch, West Grove, PA, USA) for 1 h at room temperature. After washing, protein bands on the membranes were detected with enhanced chemiluminescence (ECL) detection reagents (Thermo Fisher Scientific) using an Amersham™ Imager 680 (GE Healthcare Life Sciences, Marlborough, MA, USA).

### 2.6. Bead-Based Multiplex Flow Cytometric Analysis of Exosomal Surface Markers

The isolated ASC-exosomes were captured and labeled with the MACSPlex Exosome kit, human (Miltenyi Biotec, Bergish Gladbach, Germany) according to the manufacturer’s instructions. Briefly, 2 μg of ASC-exosomes were incubated overnight at 4 °C with 39 different capture beads. The captured exosomes were labeled with a mixture of APC-conjugated anti-CD9, -CD63 and -CD81 antibodies for 1 h at room temperature. The bead populations and APC intensities were analyzed by NovoCyte 2000 R Flow Cytometer (ACEA Biosciences, San Diego, CA, USA) and data were analyzed using the NovoExpress software (ACEA Biosciences). Background was corrected with median intensity of anti-CD9-, anti-CD63-, and anti-CD81-APC signals. Assays were run in triplicate on ten independent samples.

### 2.7. LC-MS/MS Analysis of Exosomal Proteins

NanoLC-MS/MS analysis was performed at the Korea Basic Science Institute (Ochang, Korea). Briefly, to perform protein digestion, 100 μg of protein extract was dissolved in 50 mM ammonium bicarbonate (pH 8.0) to a final concentration of about 1 μg/μL. The digested protein sample was desalted with the C18 tip and dried using a speed-vacuum, and then was dissolved in 20 μL water containing 0.1% formic acid for LC-MS/MS analysis. Peptides were analyzed using an LC-MS/MS system consisting of an Easy nLC 1000 (Thermo Fisher Scientific) and an Orbitrap Fusion Lumos mass spectrometer (Thermo Fisher Scientific) equipped with a nano-electrospray source (EASY-Spray Sources, Thermo Fisher Scientific). Peptides were trapped in a 75 μm × 2 cm C18 precolumn (nanoViper, Acclaim PepMap100, Thermo Fisher Scientific) before being separated on an analytical C18 column (75 μm × 50 cm PepMap RSLC, Thermo Fisher Scientific) at a flow rate of 300 nL/min. During the chromatographic separation, the Orbitrap Fusion Lumos was operated in the data-dependent mode, automatically switching between MS1 and MS2. The MS data were acquired using the following parameters: Full scan MS1 spectral (400–1600 m/z) were acquired in the Orbitrap for a maximum ion injection time of 100 ms at a resolution of 120,000 and an automatic gain control (AGC) target value of 4.0 × 10^5^. MS2 spectra were acquired in the Orbitrap mass analyzer at resolution of 30,000 with high energy collision dissociation (HCD) of 27% normalized collision energy and AGC target value of 5.0 × 10^4^ with maximum ion injection time of 54 ms. Previously fragmented ions were excluded for 30 sec.

MS/MS spectra were analyzed using the following software analysis protocols with the Uniprot human DB. The reversed sequences of all proteins were appended into the database for calculation of false discovery rate (FDR). ProLucid was used to identify the peptides, a precursor mass error of 5 ppm, and a fragment ion mass error of 600 ppm. The output data files were filtered and sorted to compose the protein list using the DTASelect (The Scripps Research Institute, San Diego, CA, USA) with two and more peptides assignments for a protein identification and a false positive rate less than 0.01 (Supplementary File 1).

### 2.8. Lipidomics Analysis

Lipidomics analysis was performed at Creative-Proteomics (Shirely, NY, USA). Samples on dry ice were combined with 740 μL 75% methanol and 0.5 mm zirconium oxide beads. Samples were homogenized in a Bullet Blender tissue homogenizer and extracted with 270 μL chloroform. After vortexing and centrifugation, the supernatant was collected and precipitated proteins were re-extracted as detailed above. Pooled extracts were dried overnight in a speedvac, washed with 10 mmol/L aqueous ammonium bicarbonate, and then dried again in a speedvac. Samples were resuspended in 200 μL of isopropanol containing 0.01% butylated hydroxytoluene. Aliquots of each cellular lipid extract were diluted ten-fold in isopropanol:methanol (2:1, *v:v*) containing 20 mmol/L ammonium formate and 0.5 μmol/L dimyristoyl PC as a normalization standard. Aliquots of each exosome lipid extract were analyzed without dilution, after spiking aliquots of exosome lipids with 0.5 μmol/L dimyristoyl PC and 1/10 volume of 200 mmol/L ammonium formate in methanol.

For global lipidomics analysis, full scan MS spectra at 100,000 resolution (defined at m/z 400) were collected on a Thermo Scientific LTQ-Orbitrap Velos mass spectrometer in both positive and negative ionization modes. Scans were collected from m/z 200 to m/z 2000. For each analysis, 5 μL of sample was directly introduced by flow injection (no LC column) at 1 μL/min using a HESI ion source equipped with a low-flow ESI needle. The sample and injection solvent were 2:1 (*v:v*) isopropanol: methanol containing 20 mmol/L ammonium formate. The conditions were set as follows: the spray voltage was 4.0 kV, the ion transfer tube temperature was 200 °C, the S-lens value was 50%, and the ion trap fill time was 100 ms. Following MS data acquisition, offline mass recalibration was performed with the Thermo Xcalibur software according to the manufacturer’s instructions, using the theoretical computed masses for the internal standards (PC 28:0) and several common lipid species. MS/MS confirmation of ions of interest were performed using higher-energy collisional dissociation (HCD) MS/MS at 100,000 resolution and a normalized collision energy of 25 for positive ion mode, and 60 for negative ion mode. Lipids were identified using the Lipid Mass Spectrum Analysis (LIMSA) v.1.0 software linear fit algorithm, in conjunction with an in-house database of hypothetical lipid compounds, for automated peak finding and correction of 13C isotope effects. Relative quantification of abundance between samples was performed by normalization of target lipid ion peak areas to an internal standard (PC 28:0) representative of the lipid class of interest. For this untargeted analysis, no attempt was made to correct for differences in lipid species ionization due to the length or degree of unsaturation of the esterified fatty acids.

### 2.9. Animal Experimental Protocols and Functional Studies

All animal procedures were approved by the Institutional Animal Care and Use Committee (IACUC) of Hallym University and performed in accordance with its guidelines. Female SKH-1 mice, aged 6 to 8 weeks, were sensitized with topical applications with 50 μL 2% Oxazolone (Ox). After 1 week, mice were treated topically with 50 μL 0.25% Ox to both flanks once every other day for an additional 7 weeks (total of 21 exposures). After the 8th exposure, when the phenotype of AD-like chronic allergic dermatitis was established, the therapeutic effects of ASC-exosomes were evaluated as follows: 8 h after every Ox exposure, ASC-exosomes (100 μL) at doses of 1, 3, and 10 μg/head, or 0.1% dexamethasone were applied subcutaneously or topically, respectively, thrice a week for 4 weeks (total of 12 treatments). Topical dexamethasone, a type of glucocorticoid with proven efficacy in human AD, served as a positive control. Basal trans-epidermal water loss (TEWL) was measured with a TM300 connected to MPA5 (Courage+Khazaka, Cologne, Germany), and SC hydration, assessed as capacitance, was measured with a Corneometer CM820 (Courage+Khazaka). Skin or blood samples were collected 48 h after the 12th injection of ASC-exosomes.

### 2.10. Histopathological Analyses

At the end of the study period, the dorsal skin lesions of each mouse were removed, fixed with 10% neutral-buffered formalin, and embedded in paraffin. Five micrometer-thick sections were stained with hematoxylin and eosin (H&E) and toluidine blue to detect epidermal thickness and mast cells, respectively. All samples were observed using an inverted microscope (Nikon Eclipse Ti, Nikon, Tokyo, Japan) and data were representative of five observations.

### 2.11. ELISA for Pro-Inflammatory Mediators

Quantification of cytokines in tissues and IgE in serum was performed using mouse enzyme-linked immunosorbent assay (ELISA) kits in accordance with the manufacturer’s instructions for IL-4, IL-5, IL-13, TNF-α, IFN-γ, IL-17, and TSLP (Thermo Fisher Scientific), as well as for IgE (Koma Biotech, Seoul, Korea).

### 2.12. Quantifications of Ceramides and its Metabolites

Epidermal strips were harvested and lysed in RIPA buffer followed by extraction of sphingolipids, as previously reported [26,27,28]. The extracted lipids dried using a vacuum system (Vision, Daejeon, Korea) were re-dissolved in methanol and analyzed by LC-ESI-MS/MS (API 3200 QTRAP mass, AB SCIEX, MA, USA) by the multiple reaction mode (MRM) [27]. The ceramide MS/MS transitions (m/z) were 510→264 for C14-ceramide, 538→264 for C16-ceramide, 566→264 for C18-ceramide, 594→264 for C20-ceramide, 622→264 for C22-ceramide, 648→264 for C24:1-ceramide, and 650→264 for C24-ceramide. The dihydroceramide MS/MS transitions (m/z) were 512→284 for C14-dihydroceramide, 540→284 for C16-dihydroceramide, 568→284 for C18-dihydroceramide, 596→284 for C20-dihydroceramide, 624→264 for C22-dihydroceramide, 650→284 for C24:1-dihydroceramide, and 652→284 for C24-dihydroceramide. The sphingoid bases MS/MS transitions (m/z) were 286→238 for C17 sphingosine, 366→250 for C17 sphingosine 1-phosphate (S1P) as an internal standard, 300→252 for C18 sphingosine, and 380→264 for C18 S1P. Data were acquired using Analyst 1.5.1 software (Applied Biosystems, Foster City, CA, USA). The levels of each ceramide subspecies were quantitated using calibration curves with the various concentrations of analytes and internal standard ratio and expressed as pmol/g wet weight of epidermis.

### 2.13. Enzyme Activity Assays for Sphk1 and S1PL

Activities of sphingosine kinase (Sphk) 1 or S1P lyase (S1PL) were assessed as described previously [29,30,31]. Briefly, lipid extractions were performed by the addition of 100 pmol C17-sphinganine-1-phosphate or 100 pmol of (2E)-d5-hexadecenal, respectively. The extracted lipids were dried using a vacuum system (Vision), to then be re-dissolved in methanol and analyzed by an LC-ESI-MS/MS (AB SCIEX), as described previously [31,32]. Data were acquired using Analyst 1.5.1 software (Applied Biosystems, Foster City, CA, USA). The activity of Sphk1 or S1PL was expressed as S1P or pentadecanal, respectively (pmol per mg of protein per min).

### 2.14. Electron Microscopy

The ultrastructural analysis by electron microscopy (EM) was performed as previously described by the EM facility of UCSF Dermatology under their experimental protocol [33,34,35]. Briefly, skin biopsy specimens were fixed in half-strength Karnovsky fixative to assess the delivery and secretion of lamellar body contents. Following standard fixation, tissue samples were post-fixed in reduced osmium tetroxide before epoxy embedding. The samples were cut on a Leica Ultracut E microtome (Leica Microsystems, Wetzlar, Germany) and imaged on a JEOL 100CX transmission electron microscope (JEOL, Tokyo, Japan) using a Gatan Bioscan Camera (Gatan).

### 2.15. Analysis of RNA-Sequencing Data

Quantification of RNAs was performed using the Quant-IT RiboGreen (Invitrogen, Karlsruhe, Germany). To check the quality of RNAs, DV200 value (% of RNA fragments > 200 nucleotides) was determined by using a Bioanalyzer 2100 (Agilent Technologies, Santa Clara, CA, USA) and DV200 value of RNAs obtained from 12 samples was > 64%. A 100 ng of total RNA was subjected to a sequencing library construction using the SureSelect RNA Direct Library Preparation kit (Agilent Technologies) according to the manufacturer’s protocol. To capture mouse exonic regions, the SureSelect XT Mouse All Exon Kit (Agilent Technologies) was used according to the standard Agilent SureSelect Target Enrichment protocol. A 250 ng of cDNA library was mixed with hybridization buffers, blocking mixes, RNase block, and 5 µl SureSelect all exon capture library. Hybridization to the capture baits was conducted at 65 °C using heated thermal cycler lid option at 105 °C for 24 h in a PCR thermocycler. The captured library was then washed and subjected to a second round of PCR amplification. The final purified product was then quantified using qPCR according to the qPCR Quantification Protocol Guide (KAPA Library Quantification kits for Illumina Sequencing platforms) and qualified using the TapeStation DNA ScreenTape D1000 (Agilent Technologies). Sequencing of libraries was carried out using a NovaSeq 6000 (Illumina, San Diego, California, USA) with 100 nt paired-end reads.

After removing low quality and adapter sequences, the processed reads were aligned to the reference genome sequence of Mus musculus (mm10) using the HISAT v2.1.0 [36]. Transcript assembly and abundance estimation were conducted using the StringTie [37]. Genes with one more than zeroed read count values in the samples were excluded and 1 was added to each read count value of filtered genes to facilitate log2 transformation. Filtered data were log2-transformed and subjected to trimmed mean of M values (TMM) normalization [38]. A total of 10,369 genes were identified in 12 samples and all of these genes and processed data were listed in Supplementary File 2. Statistical significance of the differential expression data was determined using exactTest on edgeR [39]. For differential expressed gene (DEG) set, hierarchical clustering analysis was performed using complete linkage and Euclidean distance as a measure of similarity.

For the identification of enriched transcriptomic signatures, gene ontology (GO) analysis of DEGs was carried out using DAVID 6.8 (https://david.ncifcrf.gov). The *p*-value was calculated using right-sided hypergeometric tests.

### 2.16. Statistical Analysis

Data were analyzed using the Prism 8.0 (GraphPad Software Inc., San Diego, CA, USA). The comparison among different groups was performed by one-way analysis of variance (ANOVA) followed by a Dunnett’s multiple-comparison of multiple means. All values are expressed as mean ± SEM. *p*-values of * *p* < 0.05; ** *p* < 0.01, and *** *p* < 0.001 were considered statistically significant.

## 3. Results

### 3.1. Isolation and Characterization of ASC-Exosomes

**Figure 1:**
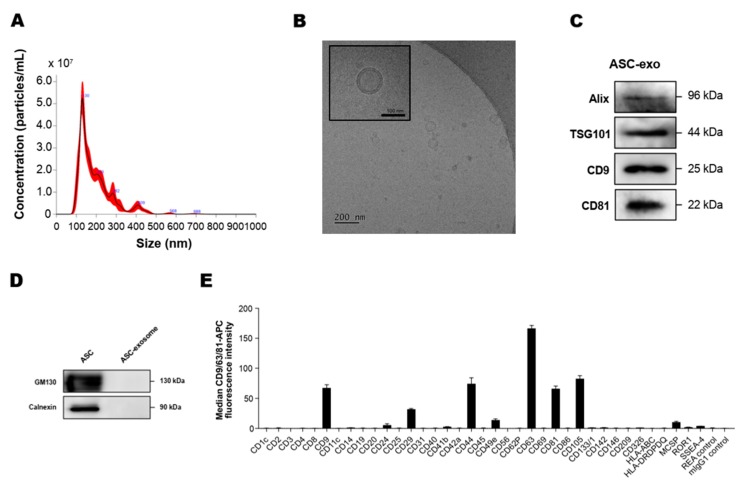
Characterization of adipose tissue-derived MSCs (ASC)-exosomes. (**A**) Representative histogram of particle concentration and size distribution of ASC-exosomes measured by nanoparticle tracking analysis (NTA). (**B**) Representative Cryo-TEM image of ASC-exosomes. Scale bar: 100 nm. (**C**) ASC-exosomes were analyzed using western blots for the presence of exosomal markers such as Alix, TSG101, CD9, and CD81. (**D**) ASC-exosomes were analyzed by western blotting for the negative markers of exosomes such as GM130 and Calnexin. Abbreviation: ASC, human adipose tissue-derived mesenchymal stem cell. (**E**) Surface signature of ASC-exosomes quantified by MACSPlex Exosome Kit (human) in conjunction with flow cytometry. Data indicate APC median signal intensities of ASC-exosomes incubated with the 39 capture beads and stained with a mixture of CD9-, CD63-, and CD81-APC antibodies. Background was corrected by subtraction of median fluorescence APC intensity. *n* = 10. MW, molecular weight.

To analyze the concentration and size of ASC-exosomes, we first measured the particle concentration and size distribution using an NS300 (NTA) instrument. The results indicated that mode size of ASC-exosomes is around 130 nm (Figure 1A). Next, we performed a cryo-TM analysis to identify the structure and morphology of ASC-exosomes. Cryo-EM images revealed the spherical shape of ASC-exosomes than ranged from 30 to 200 nm in diameter and confirmed the presence of clearly discernible lipid bilayer structures as expected (Figure 1B). To further characterize the ASC-exosomes, immunoblotting was used to test the presence of markers known to be enriched in exosomes such as Alix, TSG101, CD9, and CD81 (Figure 1C) as well as negative markers including GM130 and calnexin, which are not expected to be loaded in ASC-exosomes (Figure 1D). Semiquantitative multiplex bead-based flow cytometric analysis further detected the surface expression of various CD molecules including CD63, CD81, CD9, CD105, CD44, and CD29 on ASC-exosomes (Figure 1E). As reported in previous studies on MSC-exosomes [40], no detectable expression of human leukocyte antigens (HLA-ABC and HLA-DRDPDQ) was observed on ASC-exosomes (Figure 1E). Indeed, previous reports have shown the absence of these human major histocompatibility complex (MHC) proteins or co-stimulatory molecules such as CD80 and CD86 in MSC-exosomes [41]. These results suggest that ASC-exosomes had little to no immunogenicity in allogeneic therapy.

### 3.2. Profiling of Proteins and Lipids in ASC-Exosomes

**Figure 2:**
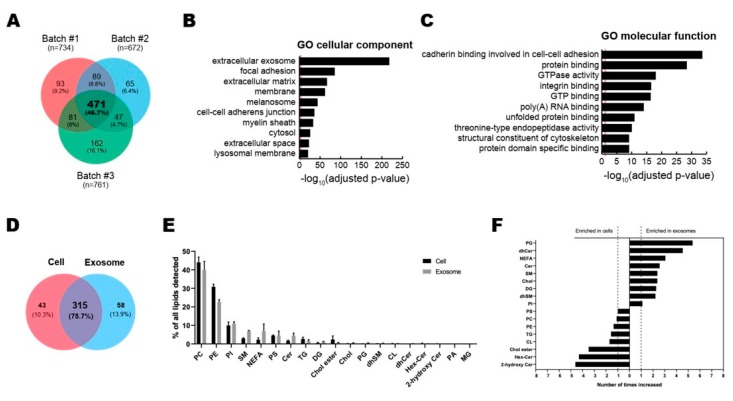
Multi-layer omics analysis of ASC-exosomes. (**A**–**C**) Proteomic analysis of ASC-exosomes by LC-MS/MS in three independent batches of ASC-exosomes. (**A**) Venn diagram showing the number of exosomal proteins that were either unique or overlapping in each indicated batch. (**B**,**C**) Gene Ontology (GO) analysis of 471 commonly found exosomal proteins. Enrichment of GO molecular function and cellular component performed using DAVID Bioinformatics resources 6.8. Vertical red lines represent the cutoff for significance of -log10(*p*-value) with Benjamini-Hochberg correction. (**D–F**) Lipidomics of ASC-exosomes. (**D**) Lipid composition of ASC-exosomes and their originating cells. A-Venn diagram showing the number of species quantified in this study. (**E**) Lipid classes detected in ASCs and ASC-exosomes. (**F**) Enrichment of lipid classes in ASCs or ASC-exosomes calculated as mol% of lipids in these samples. PC: phosphatidylcholine, PE: phosphatidylethanolamine, PI: phosphatidylinositol, SM: sphingomyelin, NEFA: non esterified fatty acid, PS: phosphatidylserine, CER: ceramide, TG: triacylglycerol, DG: Diacylglycerol, Chol ester: cholesterol ester, Chol: Cholesterol, PG: phosphatidylglycerol, dhSM: dihydrosphingomyelin, CL: cardiolipin, dhCER: dihydroceramide, Hex-Cer: hexosylceramides, PA: phosphatidic acid, MG: monoradylglycer.

To analyze the contents of ASC-exosomes, we performed the multi-omics analysis with 3 batches of ASC-exosomes for proteins and lipids (Figure 2). Proteomic analysis by LC-MS/MS revealed a total of 1008 proteins in ASC-exosomes and common 471 proteins (46.7%) among 3 batches of ASC-exosomes (Figure 2A and Supplementary File 1). GO analysis showed 471 commonly found exosomal proteins associated with “extracellular exosome” localization (Figure 2B). The molecular functions on GO terms were highly associated with binding capacities including cadherin in cell-cell adhesion, protein, integrin, GTP, poly(A) RNA, unfolded protein, and protein domain (Figure 2C).

A total of 358 and 373 lipid species were quantified in ASC-exosomes and their originating ASCs, respectively, bringing about 315 species common for the two samples (Figure 2D). The quantified molecular lipid species belong to 19 different lipid classes (Figure 2E). The overall lipid composition of ASCs and ASC-exosomes are similar, but the ratios of sphingolipids and non-esterified fatty acids (NEFA) to total lipids are increased in ASC-exosomes (Figure 2F). The ratios of sphingolipids are 5% in ASCs and 12% in ASC-exosomes, whereas the ratios of NEFAs are 2% in ASCs and 7% in ASC-exosomes.

### 3.3. ASC-Exosomes Improve Atopic Dermatitis (AD) in Ox-Induced Chronic Dermatitis Model

**Figure 3:**
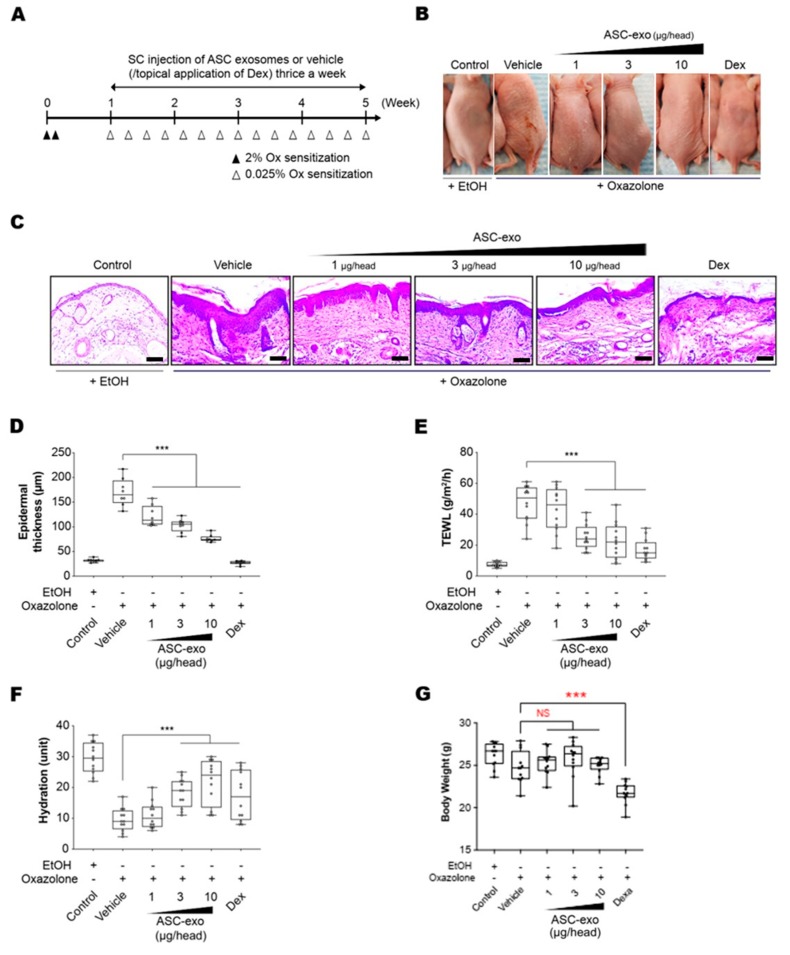
ASC-exosomes improve atopic dermatitis (AD) induced by repeated exposure to Ox. (**A**) Schematic diagram of the study protocol. The first two days 2% Ox was applied, before leaving 5 days for recovery. Dexamethasone was topically applied thrice a week as a positive control. (**B**) Representative dorsal skin photographs of each treatment group showing comparison of AD-like skin lesions (**C**) Representative images of hematoxylin and eosin (H&E)-stained epidermal histological sections from dorsal skin of mice from different treatment groups showing thickness of epidermis. The sections were visualized with a 20x magnification. Box and whisker plot of (**D**) epidermal thickness, (**E**) TEWL, and (**F**) stratum corneum (SC) hydration. The median and the 5th and 96th percentile are shown. Error bars depict the SEM. ***p < 0.001 vs. vehicle. (**G**) Effects of ASC-exosomes and dexamethasone on body weight changes. Note that the treatment of dexamethasone induced significant body weight loss. *n* = 12 per group. ***p < 0.001 determined by one-way ANOVA.

To determine the therapeutic potential of ASC-exosomes, we used SKH-1 mice that were exposed to multiple Ox treatments (Figure 3A), which progress to a model of allergic contact dermatitis. These mice exhibited diverse features of human AD, including permeability barrier abnormality (Figure 3B). ASC-exosomes treatment markedly reduced the Ox-induced epidermal hyperplasia and visible erythema (Figure 3B) which were paralleled with significant reductions in epidermal thickness (Figure 3C,D). We further confirmed the effect of ASC-exosomes by measuring TEWL and stratum corneum (SC) hydration that were significantly impaired by multiple Ox-exposures (Figure 3E,F). In the control group, the TEWL was approximately 15 g/(m2h) throughout the experiment (Figure 3E). However, the TEWL was significantly increased (~50 g/(m2h) in response to repeated Ox-exposure (Figure 3E). The increase was dose-dependently blunted depending on the ASC-exosomes treatment (Figure 3E). Additionally, ASC-exosomes also normalized the decreased SC hydration observed in vehicle-treated group (Figure 3F). Although dexamethasone also improved the epidermal thickness, TEWL, and SC hydration, it showed marked loss of body weight (Figure 3G). On the contrary, ASC-exosomes had no significant effect on the body weight.

### 3.4. ASC-Exosomes Decrease Skin Inflammation in AD Mice

**Figure 4:**
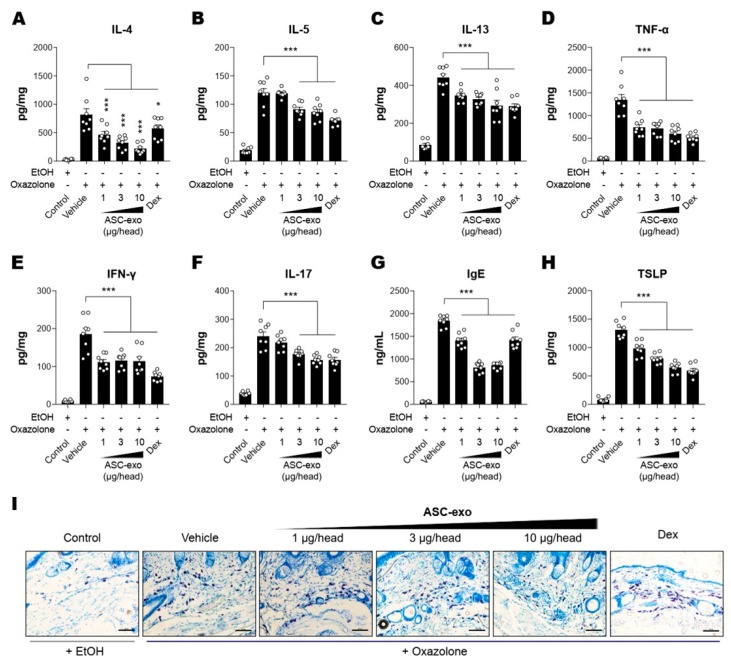
ASC-Exosomes reduce the level of multiple inflammatory cytokines in AD-like skin lesion of SKH-1 mice. The concentrations of (**A**) IL-4, (**B**) IL-5, (**C**) IL-13, (**D**) TNF-α, (**E**) IFN-γ, (**F**) IL-17, (**G**) IgE, and (**H**) TSLP in serum or lesional skin tissues were detected by ELISA. (**I**) Toluidine blue staining of the dorsal skin sections showing infiltration of mast cells into the AD-like skin lesions. Error bars depict the SEM. *n*  =  8. *: *p*  <  0.05, **: *p*  <  0.01, ***: *p* < 0.001 vs. vehicle. Dex: dexamethasone.

It has been demonstrated that epidermal barrier disruption drives skin inflammation, leading to increased production of pro-inflammatory cytokines (“outside to inside” hypothesis) [42]. Hence, we next assessed whether ASC-exosomes could decrease cytokine production in Ox-induced AD mice. The results showed that constitutive levels of cytokines including IL-4, IL-5, IL-13, TNF-α, IFN-γ, or IL-17 were relatively low in the SC of untreated control mice, whereas they were dramatically elevated in lesional SCs of AD mice that were repeatedly exposed to Ox (Figure 4A–F). ASC-exosomes treatment significantly decreased the production of all cytokines in a dose-dependent manner (Figure 4A–F). Among the multiple cytokines produced in AD skin, Th-2 cell-mediated increase in cytokines such as IL-4, IL-5, IL-13, and TNF-α were reported to trigger IgE production in B cells [43,44,45], followed by mast cell infiltration into dermis in order to facilitate allergic responses. Accordingly, we next examined whether ASC-exosomes modulate IgE synthesis and mast cell infiltration into dermis. Consistently with our previous study [24], significant increases in IgE production and IgE-induced increase in mast cell infiltration were observed in AD model mice, whereas treatment of ASC-exosomes markedly reduced these allergic inflammation responses (Figure 4G,I).

TSLP, an epithelial-derived IL-7-like cytokine, has been implicated in the pathogenesis of AD [46]. It has been demonstrated that the expression level of TSLP in lesional SCs correlates with severity of AD both in human patients and animal models [47,48]. While Th2 cytokines and IgE stimulates TSLP production, TSLP also acts as an accelerator of Th2 cytokines [49], indicating an autocrine amplification loop of inflammatory reaction in skin. Our ELISA results showed that ASC-exosomes significantly reduced the TSLP production in a dose-dependent manner (Figure 4H). Since TSLP is also known as a major inducer of pruritus [50], this result implied ASC-exosomes helped itching reduction.

### 3.5. ASC-Exosome-Mediated Improvements in Epidermal Barrier Functions Might be Attributed to Increased Production of Epidermal Ceramides

**Figure 5:**
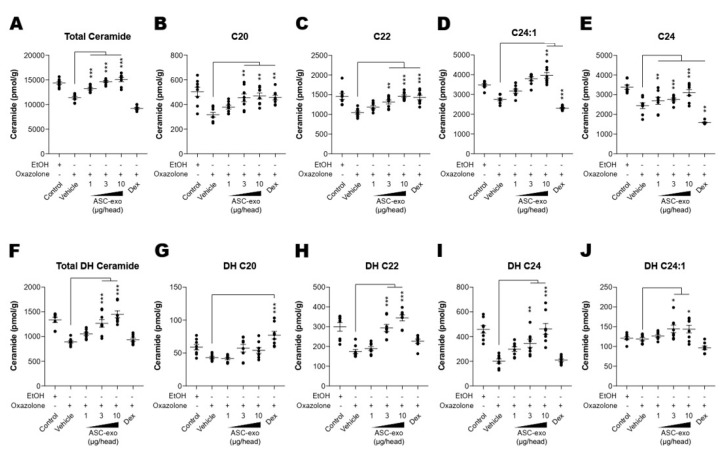
ASC-exosomes increase the level of ceramides in skin lesions. (**A–J**) Mass spectrometry analysis of ceramide and dihydroceramide species. Lipids were extracted from the homogenate of dorsal skins corresponding to 1 mg protein. *n* = 8 per group. Error bars depict the SEM. *: *p*  <  0.05, **: *p*  <  0.01, ***: *p* < 0.001 vs. vehicle. Dex: dexamethasone.

Both synthesis and secretion of epidermal lipids, in particular ceramide, from keratinocytes into the extracellular matrix of SC are required to form the epidermal permeability barrier [51]. To obtain additional insight into the basis of the protective effects seen in response to ASC-exosomes, we next determined whether ASC-exosomes enhance ceramide synthesis. Quantification of lipids using liquid chromatography-tandem mass spectrometry (LC-MS/MS) showed a significant increase in production of total ceramide after treatment with ASC-exosomes (Figure 5A). While ceramides are composed of a sphingoid base (sphingosine) linked to different chain lengths of fatty acid, a significant decrease in long-chain ceramides (above C20:0) in AD skin has been highlighted in previous studies [52,53,54]. Therefore, we further clarified the change in long chain ceramides, and our LC-MS/MS analysis revealed that ASC-exosomes selectively increase the number of ceramides carrying long acyl chains, C20:0, C22:0, C24:0, and C24:1 (Figure 5B–E). To assess a possible mechanism underlying this ceramide synthesis, we next determined changes in production of dihydroceramides, a precursor lipid that is transiently produced during de novo ceramide synthesis [55]. Similar to acyl chain patterns of ceramide, long-chain dihydroceramides, except for C20:1, were significantly enhanced in AD skin following ASC-exosomes treatment (Figure 5F–J).

**Figure 6:**
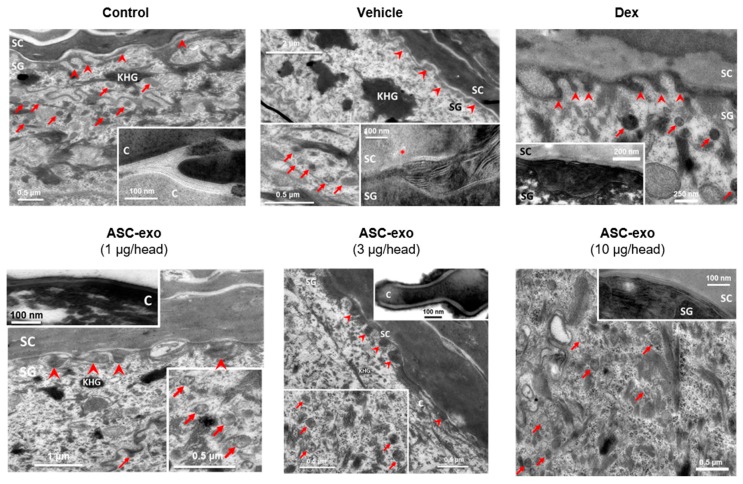
ASC-exosomes restore oxazole-induced defects in the lamellar secretory system. Electron micrographs of epidermis. Inserts show high magnification of lamellar bodies and/or processed lamellar lipids as indicated. Note that lamellar bodies are more abundant at the SG-SC interface in the ASC-exosomes treated group compared with the vehicle and dexamethasone groups. KHG: keratohyaline granules, C: corneocyte, arrows: lamellar bodies, arrow heads: lipid secretion points, *: lipid phase separation (indicating incomplete processing). Scale bar: 100 nm.

Newly produced ceramides are packed and stored within epidermal lamellar bodies, which are then secreted at the interface of stratum granulosum (SG) and SC to form lamellar lipid sheets that comprise the epidermal permeability barrier [51]. In human AD, decreased SC ceramide content and altered lamellar lipid structure are known to be associated with skin barrier abnormalities [56,57]. Consistent with this, we observed numbers of lamellar bodies (arrows) and defective processing of secreted lamellar lipids (noted by *) in oxazolone-treated mice (Figure 6, “Vehicle”) versus control mice. These defects were completely reversed by treatment with ASC-exosomes. In contrast, dexamethasone treatment was associated with improved lipid processing but few lamellar bodies, consistent with our finding that this treatment reduced epidermal ceramide content.

Taken together, these results suggest that ASC-exosomes stimulates epidermal ceramide production/secretion and the formation of lamellar bilayers at the SG–SC interface, leading to improvements in barrier function.

### 3.6. ASC-Exosomes Normalize Altered Gene Expression in Ox-Induced Skin Lesions

**Figure 7:**
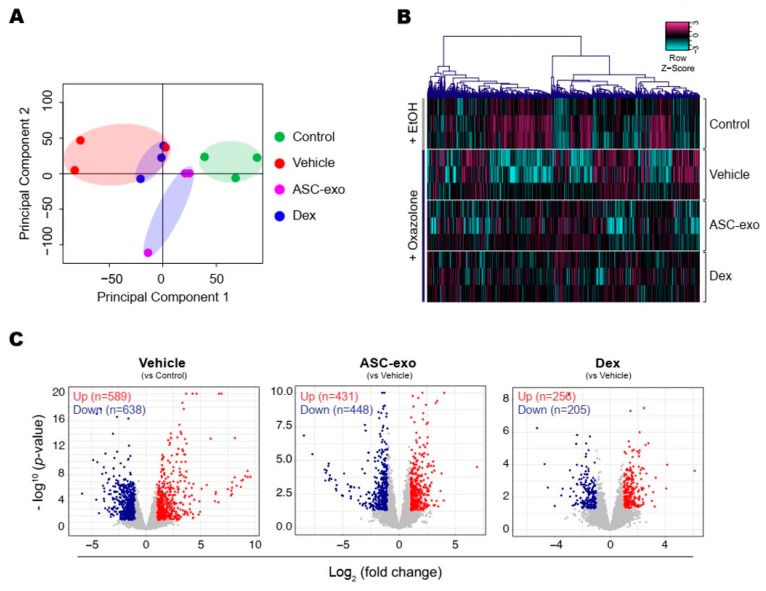
ASC-exosomes modulate distinct gene expression program during AD pathogenesis. (**A**) The principal component analysis (PCA) showing the first two principal components of RNA-Seq data regarding their correlation. (**B**) Heatmap showing 1802 differential expressed gene (DEGs). (**C**) Volcano plot shows the DEGs in vehicle, ASC-exosomes-, or dexamethasone-treated group. Red dots indicate significantly up-regulated genes and blue dots indicate down-regulated genes.

To identify the cellular pathways modulated by ASC-exosomes and decipher the mechanisms that contributed to skin barrier restoration, we performed high-throughput RNA sequencing on skin lesions from control, vehicle-, ASC-exosomes- (SC, high), and dexamethasone-treated animals. As shown in Figure 7A, the principal component analysis (PCA) showed a clear separation of ASC-exosomes-treated group from control, vehicle, or dexamethasone-treated group, indicating that ASC-exosomes regulate distinct gene expression program during recovery from the AD. Using a threshold of a minimum |fold change| > 2 (*p* < 0.05), we identified a total of 1802 DEGs in epidermis of each animal group (Figure 7B,C).

**Figure 8:**
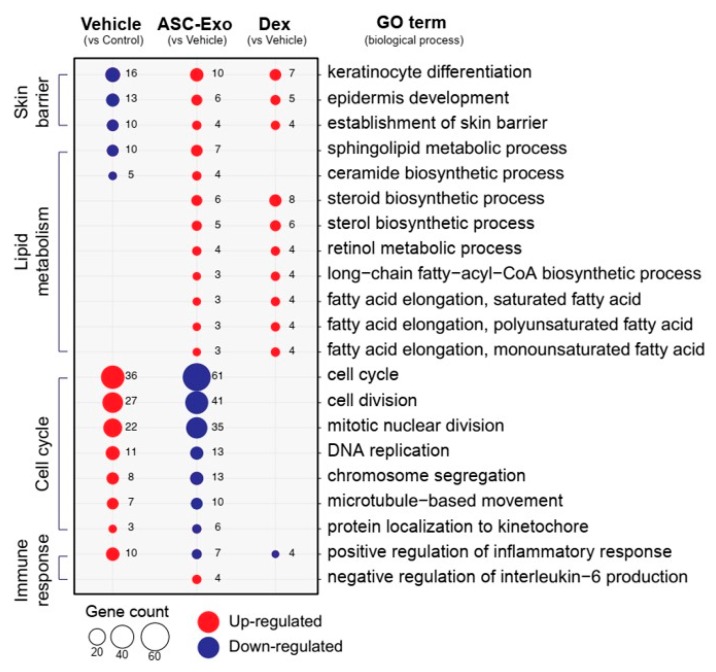
ASC-exosomes normalize altered gene expression in a murine model of allergic dermatitis. Bubble plot shows the significantly (*p* < 0.05) altered GO biological processes in vehicle, ASC-exosomes-, and dexamethasone-treated groups compared with the indicated control group. The size of the bubble is proportional to the number of genes. Red bubbles indicate up-regulation and blue bubbles denote down-regulation. GO analysis was performed using DAVID bioinformatics resources 6.8 (https://david.ncifcrf.gov).

**Figure 9:**
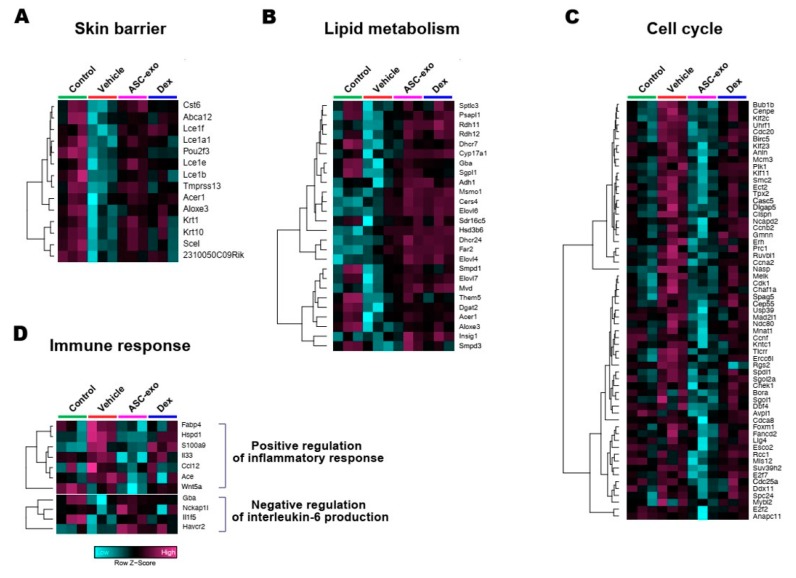
Heat map of DEGs between vehicle and ASC-exosomes that mapped to the indicated GO in Figure 8. (**A**) Skin barrier. (**B**) Lipid metabolism. (**C**) Cell cycle. (**D**) Immune response.

We next performed gene ontology analysis to further define major biological processes driving the ASC-exosomes-mediated recovery of epidermal barrier functions and ultimate alleviation of AD. The results showed that SC delivery of ASC-exosomes normalized the expression of genes that were significantly down-regulated in the vehicle group, especially those involved in the epidermal barrier functions such as keratinocyte differentiation, epidermis development, and establishment of skin barrier (Figure 8 and Figure 9). Lipid metabolic processes such as ceramide biosynthetic and sphingolipid metabolism were also significantly de-repressed by ASC-exosomes treatment, indicating that ASC-exosomes improve skin barrier functions by activating lipid metabolism in lesional SC in AD. Furthermore, ASC-exosomes down-regulated the expression of genes that were activated in the vehicle group, which were related to biological functions such as cell cycle and positive regulation of inflammatory responses. Dexamethasone only partly reversed the Ox-induced changes in gene expression.

Collectively, these data demonstrate that subcutaneous administration of ASC-exosomes normalized global epidermal gene expression changes that were abnormally altered during AD pathogenesis.

## 4. Discussion

Given our previous finding that ASC-exosomes alleviates AD-like symptoms in murine model of AD by suppressing systemic inflammation [24], in the present study, we investigated whether ASC-exosomes could restore epidermal permeability barrier (Figure 5 and Figure 6) that is significantly impaired in AD mice. The results of this study indicate that treatment of ASC-exosomes lead to a significant improvement in epidermal barrier functions by de-repressing synthesis of ceramides and dihydroceramides carrying long acyl chains (Figure 5) and inducing the formation and secretion of lamellar bodies at the SG-SC interface (Figure 6). Transcriptomic analysis of AD-like lesional skins showed that treatment of ASC-exosomes normalizes the altered gene expressions (which involves multiple biological functions such as skin barrier maintenance, lipid metabolism, cell cycle, and immune responses) by repeated exposure of Ox (Figure 7 and Figure 8). These findings suggest an important and unprecedented mechanism of ASC-exosome to preserve epidermal barrier function in the face of AD.

**Figure 10:**
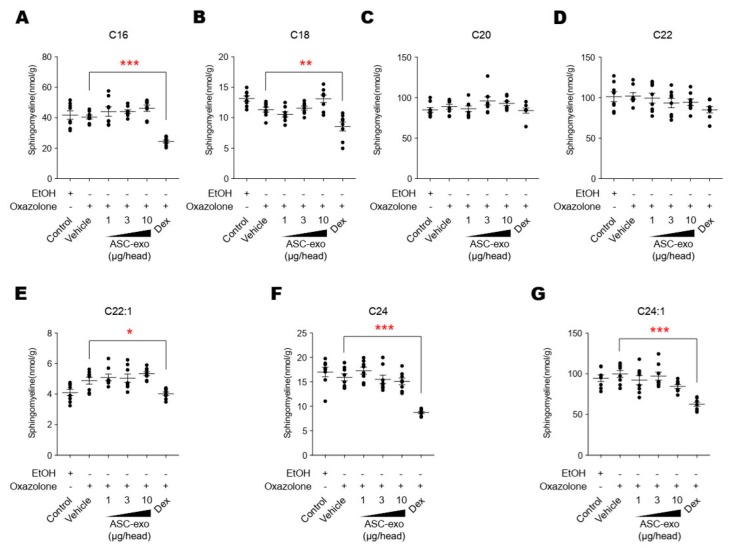
ASC-exosomes do not affect the sphingolipid hydrolysis pathway. (**A–G**) The lipid profiles were determined by LC-MS/MS measurements. Note that dexamethasone treatment significantly reduces the levels of sphingomyeline except for those with C18 and C20 acyl chains. Error bars depict the SEM. *n*  =  8. *: *p* < 0.05, **: *p*  <  0.01, ***: *p* < 0.001 vs. vehicle. Dex, dexamethasone.

**Figure 11:**
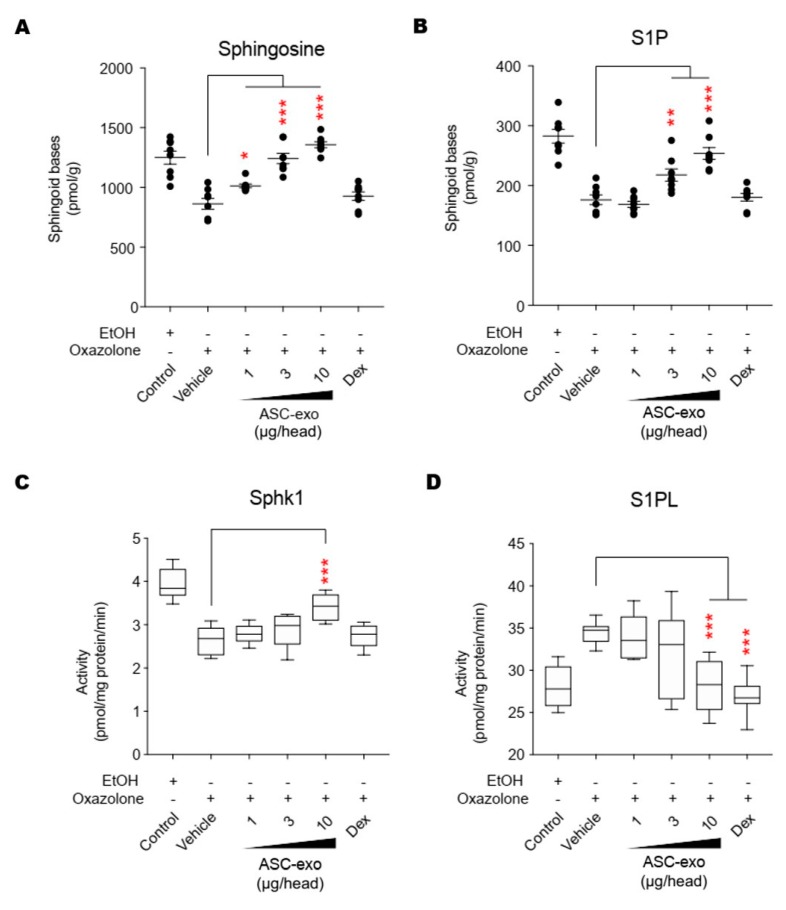
ASC-exosomes regulate the salvage pathway to increase ceramides in skin lesion. (**A**–**D**) The levels of sphingosine and S1P, and the activities of Sphk1 and S1PL were determined by LC-MS/MS measurements. Error bars depict the SEM. *n*  =  8. *: *p* < 0.05, **: *p*  <  0.01, ***: *p* < 0.001 vs. vehicle. Dex, dexamethasone.

AD pathogenesis has a complex underlying mechanism, which includes changes in the skin barrier, an abnormal immune signaling, and a defective terminal keratinocyte differentiation that lead to decreased levels of ceramides, filaggrin, and antimicrobial peptides [58]. Among them, the skin barrier disruption has been known to play a role in the progression of AD, linking the structural changes with severe skin inflammation as it triggers the easy access of pathogens, allergens, and toxic environmental pollutants [59]. Ceramides, the major constituents of the lamellar barrier, has been shown to contribute greatly to the differentiation of keratinocytes, and subsequently to the epidermal barrier function [60,61]. It is well known that ceramides levels are frequently reduced in the lesional skin of patients with AD [56,57,62], as well as psoriasis [63]. A recent study with serine palmitoyltrasferase (SPT), the rate-limiting enzyme in the de novo synthesis of ceramides, in knockout mice further suggested that the decrease of ceramide levels in the epidermis leads to psoriasis-like skin inflammation [64], supporting the essential role of ceramides in epidermal barrier function. In the present study, we also found that disruption of epidermal barrier by repeated exposure to Ox is accompanied by significant reduction in ceramide levels (Figure 5). However, subcutaneous administration of ASC-exosomes could effectively increase the production of very-long acyl chain ceramide by a de novo synthesis pathway (Figure 5 and Figure 6), rather than a sphingomyelin (SM) hydrolysis pathway, by the action of sphingomyelinases (SMases) (Figure 10). In addition to the structural role of ceramides in the epidermis, ceramides and its metabolites have been recognized as signaling lipids that regulate multiple cellular/biological responses, e.g., proliferation, differentiation, and apoptosis. In particular, sphingosine-1-phosphate (S1P), a distal metabolite of ceramide, has been shown to activate anti-apoptotic activity to protect cells from ceramides-induced apoptosis, to increase cell motility, and to stimulate keratinocytes differentiation. Interestingly, ASC-exosomes induced the increased sphingoid bases including sphingosine and S1P. In the present study, the ASC-exosomes-mediated ceramide conversion beyond sphingosine to S1P is tightly regulated by two enzymes which account for regulation of cellular S1P content—that is, activating an S1P generating enzyme Sphk1, and conversely, suppression of S1PL, an S1P degrading enzyme (Figure 11). It was also found that ASC-exosomes activate certain gene expression programs such as keratinocytes differentiation-associated genes (Figure 7 and Figure 8). Although it remains elusive whether these effects are directly due to the actions of exosomal proteins, microRNAs, mRNAs, long noncoding RNAs (lncRNAs), or more than one of these molecular components, our findings indicate that ASC-exosomes stimulate production of epidermal ceramides through a de novo synthesis pathway, followed by further metabolic conversion to generate S1P (Figure 11B), contributing to enhance keratinocytes differentiation, helping form a proper epidermal permeability barrier in the AD pathophysiology.

Deep RNA sequencing analysis on the dorsal skin of AD-like lesions showed unique transcriptomic signatures regulated by ASC-exosomes in the context of AD pathophysiology. For example, ASC-exosomes significantly up-regulate genes involved in formation of skin barrier, keratinocyte differentiation, and synthesis of lipid species such as ceramides and sphingolipids. Notably, the signaling for cell cycle in AD lesions was markedly inhibited by ASC-exosome. Currently, the mechanistic basis for the cell cycle arrest by ASC-exosomes treatment remains elusive. This mechanistic basis can be elucidated if this cell cycle arrest initially arises solely from the keratinocytes as a result of ASC-exosomes treatment or there are indirect effects in response to ASC-exosomes treatment. However, according to the evidence showing that ceramides regulate keratinocyte differentiation [65], cellular growth arrest [66], and apoptosis [67] by modulating various signaling pathways such as mitogen-activated protein kinases (MAPKs), peroxisome proliferator-activated receptor gamma (PPARγ), and stress-activated protein kinase/c-Jun NH2-terminal kinase (SAPK/JNK) pathways, the observed effects on cell cycle arrest could be attributable, at least in part, to the increased synthesis of ceramides by ASC-exosomes.

Preclinical application of MSC-exosomes has been explored in various disease models [68]. Up to now, no direct evidence is available on the clinical relevance of preclinical results with exosomes derived from human cells in animal models. However, results from phase I/IIa clinical trials with human umbilical cord blood (UCB)-derived MSCs was confirmed the therapeutic efficacy of human UCB-MSCs observed in animal studies [69]. Since it has been established that the therapeutic effects of stem cells are mainly mediated by paracrine factors including exosomes, the therapeutic effects of ASC-exosomes observed in current study provide the rationale for the treatment of AD in human.

In conclusion, the present study highlights a hitherto unappreciated role of ASC-exosomes in AD, and defines a unique role by which the ASC-exosomes repair defective epidermal permeability barrier functions. ASC-exosomes appear to significantly induce de novo synthesis of ceramides and modulate the multiple gene expression program including differentiation of keratinocytes, lipid metabolism, cell cycle, and immune response. We propose that ASC-exosomes offer a promising cell-free therapeutic option for the treatment of AD, a disease which requires a systematic and multi-pronged approach.

## Supplementary Materials

The following are available online at https://www.mdpi.com/2073-4409/9/3/680/s1, Supplementary File 1. List of exosomal proteins identified by LC-MS/MS analysis. Supplementary File 2. List of transcripts identified in mouse skin lesion by RNA-Sequencing analysis.
